# Odd and Even Numbered Ferric Wheels

**DOI:** 10.1002/advs.202304553

**Published:** 2023-08-27

**Authors:** Daniel J. Cutler, Angelos B. Canaj, Mukesh K. Singh, Gary S. Nichol, David Gracia, Hiroyuki Nojiri, Marco Evangelisti, Jürgen Schnack, Euan K. Brechin

**Affiliations:** ^1^ EaStCHEM School of Chemistry The University of Edinburgh David Brewster Road Edinburgh EH9 3FJ UK; ^2^ Instituto de Nanociencia y Materiales de Aragón (INMA) CSIC & Universidad de Zaragoza Zaragoza 50009 Spain; ^3^ Institute for Materials Research Tohoku University Katahira 2‐1‐1 Sendai 980–8577 Japan; ^4^ Universität Bielefeld | Fakultät für Physik Postfach 100131 D‐33501 Bielefeld Germany

**Keywords:** Fe^III^, even numbered wheel, odd numbered wheel, magnetism, spin frustration

## Abstract

The structurally related odd and even numbered wheels [Fe^III^_11_Zn^II^_4_(tea)_10_(teaH)_1_(OMe)Cl_8_] (**1**) and [Fe^III^_12_Zn^II^_4_(tea)_12_Cl_8_] (**2**) can be synthesized under ambient conditions by reacting Fe^III^ and Zn^II^ salts with triethanolamine (teaH_3_), the change in nuclearity being dictated by the solvents employed. An antiferromagnetic exchange between nearest neighbors, *J* = ‐10.0 cm^−1^ for **1** and *J* = −12.0 cm^−1^ for **2**, leads to a frustrated *S* = 1/2 ground state in the former and an *S* = 0 ground state in the latter.

## Graphical abstract

Structurally related odd and even numbered ferric wheels, [Fe^III^_11_] and [Fe^III^_12_], can be isolated by exploiting different combinations of solvents. Both wheels display antiferromagnetic exchange leading to a frustrated *S* = 1/2 ground state in the former and an *S* = 0 ground state in the latter. High‐field magnetization data reveal a series of level crossings for both compounds.

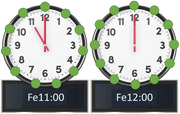

## 1 Introduction

Understanding the magnetic properties of antiferromagnetic (AF) transition metal wheels,^[^ ^1^ ^]^ whose behavior depends on both size (length) and topology is key to future application in, for example, quantum information processing^[^ ^2^ ^]^ and the study of exotic frustration effects.^[^ ^3^ ^]^ Even‐numbered homometallic, homovalent AF wheels, which are commonplace and exist in a breadth of nuclearities,^[^ ^4^ ^]^ are characterized by a diamagnetic spin ground state. Studies of these species have revealed interesting quantum phenomena, including coherent tunneling of the Néel vector,^[^ ^5^ ^]^ spin‐multiplet mixing effects,^[^ ^6^ ^]^ magnetic level repulsions, and symmetry‐related anomalies.^[^ ^7^ ^]^ There are fewer examples of antiferromagnetic homometallic, heterovalent species, but here the different valences (spins) on neighboring ions can be exploited to stabilize unusual spin ground states. An example is the [Mn^III^_6_Mn^II^_6_] (6 x *s* = 2, 6 x *s* = 5/2) wheel built with *N*‐methyldiethanolamine which has an *S* = 7 ground state.^[^ ^8^ ^]^

Odd‐numbered AF wheels present the chance to examine spin frustration effects, perhaps more commonly associated with spin glasses and solid‐state materials conforming to the kagome and pyrochlore structures.^[^ ^9^ ^]^ Frustration – here understood as competing interactions – in molecular species^[^ ^3^ ^]^ can lead to enhanced ground‐state degeneracy, low‐lying singlet states, noncollinear ground states, unusual magnetization plateaus and jumps, and attractive magnetocaloric properties.^[^ ^10^ ^]^ Odd‐numbered wheels with N > 3 (where *N* = number of metal ions), however, remain remarkably rare. A search of the Cambridge Structural Database reveals these are limited to [Cu^II^_5_],^[^ ^11^ ^]^ [V^IV^_7_],^[^ ^12^ ^]^ [Ti^IV^_9_] and [Ti_8_M^III^],^[^ ^13^ ^]^ [Cr^III^_8_M^II^]^[^ ^14^ ^]^ and [Cr^III^_9_].^[^ ^15^ ^]^ Herein, we report the synthesis and characterization of the first odd‐numbered Fe^III^ wheel, [Fe^III^_11_Zn^II^_4_(tea)_10_(teaH)_1_(OMe)Cl_8_] (**1**), and its related even‐membered analog, [Fe^III^_12_Zn^II^_4_(tea)_12_Cl_8_] (**2**), where H_3_tea is triethanolamine.

## 2 Results and Discussion

**Figure 1:**
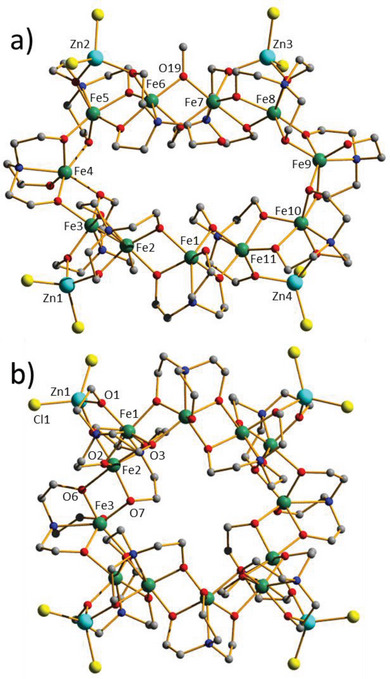
Molecular structures of complexes a) **1** and b) **2**. Color code: Fe = green, Zn = pale blue, O = red, N = dark blue, C = grey, Cl = yellow. H atoms omitted.

Reaction of FeCl_3_ and Zn(ClO_4_)_2_·6H_2_O with H_3_tea in a basic MeOH/MeCN solution (see the SI for full experimental details) leads to the formation of **1** after 3 days (**Figure** **1**). **1** crystallizes in the monoclinic space group *P*2_1_/*n* (Table S1, Figure S1), with the asymmetric unit comprising the whole cluster. The [Fe^III^_11_] metallic skeleton is somewhat S‐shaped rather than planar (Figure S2, Tables S2‐S4, Supporting Information), with all neighboring Fe ions bridged by two O‐atoms from two tea ligands, [Fe‐(μ‐O_tea_)_2_‐Fe]. The only exception to this is between Fe6‐Fe7 where one of the triethanolamine arms is protonated/nonbonded and replaced in the bridging unit by the sole μ‐OMe ligand (Fe6‐O19‐Fe7) in the cluster, [Fe‐(μ‐O_tea_)(μ‐OMe)‐Fe]. This moiety is therefore responsible for the asymmetry of the wheel, with the magnetic core of the molecule being [Fe_11_(μ‐O_tea_)_21_(μ‐OMe)_1_]. The triethanolamine ligands are of three types (Figure S3, Supporting Information). Seven are μ_4_‐bridging ([Fe_3_Zn]) with two O‐atoms bridging two Fe ions, and one O‐atom bridging between Fe–Zn. Three are μ_3_‐bridging ([Fe_3_]) with two O‐atoms bridging two Fe ions, and one O‐atom being terminally bonded. The remaining ligand is μ_3_‐bridging ([Fe_2_Zn]) with one O‐atom bridging two Fe ions, one bridging between Fe and Zn, and the third being nonbonded. The Fe‐O‐Fe angles fall within the range ≈101.6‐107.7°. The Fe ions are all six‐coordinate and in distorted octahedral geometries, while the Zn ions are four‐coordinate and tetrahedral – the remaining two coordination sites on each Zn ion being occupied by Cl ions.

The nonbonded HO‐arm of the Htea ligand is oriented towards and above the cavity of the wheel and is H‐bonded to an H_2_O molecule of crystallization (O(H)···O, ≈2.74 Å) which also has a close contact to a µ‐bridging O‐atom from a tea ligand on the inner rim of the wheel (O(H)···O, ≈3.04 Å). The monodentate O(tea) arms are also H‐bonded to H_2_O molecules of crystallization (O(H)···O, ≈2.83 Å). The latter form a near linear chain of five H_2_O molecules which also H‐bond to the terminal Cl atoms (O(H)···Cl, ≈3.4 Å). The Cl atoms in turn are also H‐bonded to CH_2_(tea) moieties on neighboring clusters (Cl···(H)C, ≈3.4 Å) and the latter to other CH_2_(tea) moieties (C(H)···(H)C, ≈3.5 Å). The result is a rather complicated network of H‐bonded clusters in the extended structure (Figure S4, Supporting Information).

Repetition of the synthetic procedure that produces **1**, but in DMF/MeCN affords **2** after 4 days (Figure 1). **2** crystallizes in the tetragonal space group *I*4_1_/*a* (see the SI for full details; Table S1, Figure S5, Supporting Information). Compound **2** is to some extent a symmetric analog of **1**, with the twelve tea ligands now of just two types: one µ_3_‐bridging ligand ([Fe_3_]) followed by two µ_4_‐bridging ligands ([Fe_3_Zn]) as the wheel is circumnavigated. The result is that the metallic skeleton becomes bowl‐ or U‐shaped rather than S‐shaped, with the magnetic core of the molecule being [Fe_12_(μ‐O_tea_)_22_] (Figure S6, Tables S5–S6, Supporting Information). In the extended structure of **2** wheels pack in a head‐to‐tail fashion along the *c*‐axis of the cell with a square of H_2_O molecules (O···O, ≈2.86 Å) sitting between the wheels mediating H‐bonding interactions to the O‐arms of the tea ligands (O···O, ≈2.63 Å). The result is the formation of tubular arrays of wheels down the *c*‐axis. In the *ab* plane closest intermolecular interactions are mediated between the Cl atoms and tea ligands (C(H)···Cl, ≈3.6 Å) resulting in a regular square grid of wheels. Overall, this leads to the aesthetically pleasing packing structure shown in Figure S7, Supporting Information.

**Figure 2:**
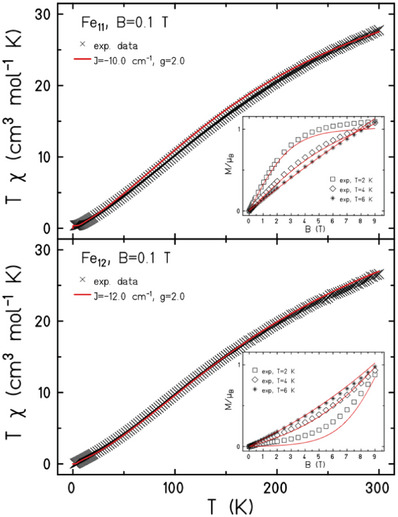
Magnetic susceptibility and magnetization data (insets) for **1** (top) and **2** (bottom). The solid red lines are simulations of the experimental data with *J* = ‐10.0 cm^−1^ and *J* = ‐12.0 cm^−1^, respectively, with *g* = 2.0. See the main text for details and Figure S8, Supporting Information, for larger versions of the insets.

The direct current (dc) molar magnetic susceptibilities, *χ*, of polycrystalline samples of **1**–**2** were measured in an applied magnetic field, *B*, of 0.1 T, over the 2–300 K temperature, *T*, range. Magnetization (*M*) data was measured in the 2–10 K and 0.5‐9.0 T, temperature and field ranges, respectively. The results are plotted in **Figure** **2** (and Figure S8, Supporting Information) in the form of the *χT* product versus *T* and *M* versus *B* (insets of Figure 2). The susceptibility data for **1** and **2** are similar, showing relatively strong AF exchange interactions between nearest neighbors with *χT* decreasing rapidly with decreasing temperature and reaching values of 0.40 and 0.05 cm^3^ K mol^−1^ at *T* = 2.0 K for **1** and **2**, respectively.

The variable‐temperature‐variable‐field magnetization data differ significantly between the two compounds. For **1** at *T* = 2 K, *M* rises rapidly with increasing field, before saturating at a value of *M* = 1.1 µ_B_. At higher temperatures, *M* rises in a more linear fashion with increasing *B*. At *T* = 2–3 K the magnetization data for **2** remains close to zero, before increasing more rapidly for fields above ≈4 T. The maximum value at *T* = 2 K and *B* = 9.0 T is just 0.88 µ_B_. With increasing temperature (4‐10 K) *M* increases in a more linear fashion with increasing field.

**Figure 3:**
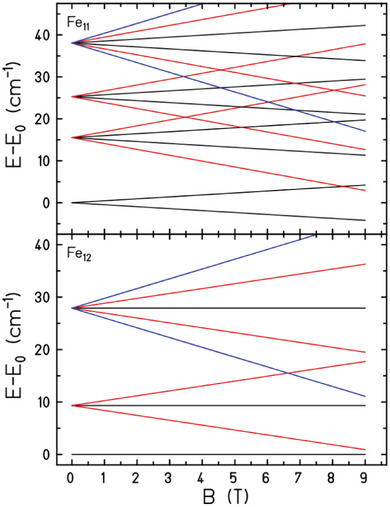
Low‐lying Zeeman energy diagram. For **1** (top) *M*  =   ± 1/2 black lines, *M*  =   ± 3/2 red lines, and *M*  =   ± 5/2 blue lines. For **2** (bottom) *M*  =  0 black lines, *M*  =   ± 1 red lines, and *M*  =   ± 2 blue lines. For **1** the levels are degenerate; the degeneracy – typically 2 – is given in Refs. ^[^ ^10^, ^16^ ^]^.

The magnetic susceptibility data can be accurately simulated using an isotropic spin‐Hamiltonian  $\hat{H}=\;-2\;\sum_{i}J_{j}{\hat{\vec{s}}}_{i}\cdot {\hat{\vec{s}}}_{j+1}$ with a coupling scheme that assumes (as a reasonable simplification) just one independent exchange interaction between nearest neighbors, *J* = −10.0 cm^−1^ for **1** and *J* = −12.0 cm^−1^ for **2**, with *g*  = 2.0 in both cases (Figure 2, red lines). The susceptibility data is also approximated well with the DFT calculated *J* values (see below), though the magnetization data is not (Figure S8, Supporting Information, blue lines). DFT suggests that *J* values vary in some range for nearest neighbor exchange interactions. Likely this leads to an incorrect approximation of ground and low‐lying excited states. The ground state for **1** is an *S* = ½ state,^[^ ^10b^ ^]^ and for **2** is *S* = 0 as can be clearly seen in **Figure** **3**. All calculations have been performed by means of the finite‐temperature Lanczos method.^[^ ^16^ ^]^

The relatively large magnitude of *J* results in sizeable level spacings between the lowest Zeeman levels (Figure 3), with the sequence of levels being as expected for odd and even spin rings.^[^ ^10^, ^17^, ^18^ ^]^ The large gap size explains the (very) small magnetization values, even in fields of up to 9 T.

**Figure 4:**
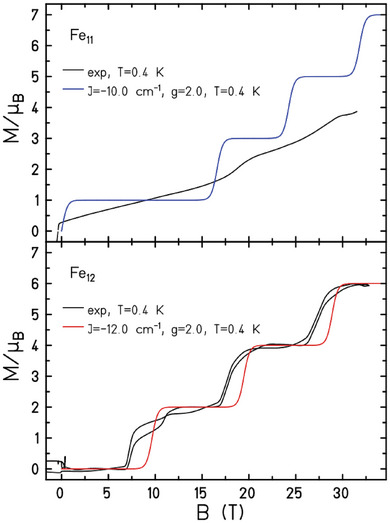
High‐field magnetization data (solid black lines for *B* > 0 and *B* < 0 T) were collected for **1** (top) and **2** (bottom) in fields up to 32.5 T. The solid blue and red lines are simulations of the experimental data with *J* = −10 cm^−1^ (**1**), −12.0 cm^‐^^1^ (**2**) at *T* = 0.4 K.

At fields of up to 32.5 T at *T* = 0.4 K the magnetization data for **1** and **2** reveal a series of step‐like level crossings (solid and dashed black lines in **Figure** **4**, Figure S8, Supporting Information). For **2** these are relatively well resolved for *B* ≈ 7.5 T (*S* = 0→1), 17.5 T (*S* = 1→2), and 27.5 T (*S* = 2→3). For **1** there appear to be two broader, less well‐resolved steps centered around *B* ≈ 18 T (*S* = 3/2→5/2) and 23 T (*S* = 5/2→7/2), the *S* = 1/2→3/2 step being smeared out. The remarkable difference between the two sets of data reflects the high and low symmetry of molecules with even and odd numbered metal ions, respectively. Specifically, for an odd‐numbered ring with lower symmetry, the local anisotropy (single ion and exchange anisotropy) is not canceled out, inducing mixing between energy levels and the broadening of magnetization steps. The steps are remarkably well simulated using the same model employed to fit the low‐field susceptibility and magnetization data, particularly for **2**, albeit slightly shifted in *M* and *B*. We attribute the small differences to a) the simplicity of the (one‐*J*) model, b) possible anisotropic contributions to the Hamiltonian relevant at very low temperatures, c) the fact that the high‐field measurement uses a pulsed field and thus may show dynamic effects such as slow equilibration and discontinuities.

**Figure 5:**
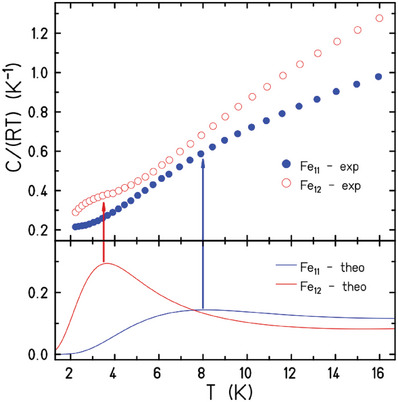
Experimental heat capacity data for **1** and **2** at zero‐applied field (top), depicted as *C/*(*RT*), where *R* is the molar gas constant. The solid blue and red lines in the bottom panel are simulations with *J* = −10 cm^−1^ (**1**), −12.0 cm^‐1^ (**2**). The arrows highlight the Schottky anomalies. The increase of the experimental data with temperature must be ascribed to the ordinary lattice contribution, which is not considered by the simulations.

This same model explains the differences observed in the heat capacity (*C*) of the two compounds (**Figure** **5**). For **1** the main feature of the zero‐applied‐field *C* is a broad Schottky anomaly centered at ≈8 K, while **2** has a more prominent Schottky anomaly at ≈3.5 K. Both features are quantitatively accounted for by the large energy gap existing between the two lowest multiplets for each respective compound (Figure 3).

To further support the relative sign and magnitude of the coupling constants above, and given that the single *J* model serves as a general/qualitative model to understand the gross features, we have performed DFT calculations (see the SI for computational details) on model complexes derived from **1** and **2** (Figures S9 and 10, Tables S7–S8, Supporting Information). All computed exchange interactions are antiferromagnetic in nature. For **1** the eleven independent coupling constants fall in the range −3.5 < *J* < −12.8 cm^−1^ (Figure S11, Supporting Information). For **2** the three independent coupling constants fall in the range −7.1 < *J* < −12.7 cm^−1^ (Figure S12). The narrower range of *J* values for **2** is consistent with the structural similarity of nearest neighbor bridging.^[^ ^19^ ^]^ DFT computed spin densities suggest a strong spin‐delocalization mechanism for the magnetic exchange interaction in both cases, with the spin on Fe^III^ centers ranging between 4.128 and 4.187 for **1** (Figure S13, Supporting Information) and 4.014–4.165 for **2** (Figure S14, Supporting Information). Both the experimentally and computationally derived *J* values agree with previous magneto‐structural correlations developed for O‐bridged Fe^III^ complexes in which the magnitude of *J* is dictated by the Fe—O–Fe angle and Fe–O distance.^[^ ^19^ ^]^

## 3 Conclusion

In summary, we have synthesized and characterized two structurally related wheels of Fe^III^, specifically the odd numbered [Fe_11_] and the even numbered [Fe_12_]. The former is the first example of an odd numbered Fe wheel. Synthetically, the formation of one over the other can be controlled via the choice of solvent, a mixture of MeCN and MeOH for the former, and a mixture of MeCN and DMF for the latter. The asymmetry in **1** originates from the presence of a protonated/nonbonded arm in one triethanolamine ligand which is replaced in the bridging unit by methoxide anion. Magnetic susceptibility, magnetization, and heat capacity measurements reveal relatively strong antiferromagnetic exchange between nearest neighbors, *J* = −10.0 cm^−1^ for **1** and *J* = −12.0 cm^−1^ for **2**. This leads to a frustrated *S* = 1/2 ground state in the former and an *S* = 0 ground state in the latter, with the relatively large value of *J* resulting in sizeable level spacings between the lowest Zeeman levels. High‐field magnetization data collected up to ≈32.5 T reveals a series of step‐like level crossings for both compounds.

## Conflict of Interest

The authors declare no conflict of interest.

## Author Contributions

D.J.C. and A.B.C. contributed equally to this work. D.J.C. and A.B.C. performed the synthesis, collected and analyzed PXRD data, and collected the magnetometry data. G.S.N. collected and solved single crystal XRD data. M.K.S. carried out the theoretical analyses. D.J.C. and H.N. measured the high‐field magnetization data. M.E. measured and analyzed the heat capacity data. J.S. fitted the magnetic data. E.K.B. conceived the idea. All authors contributed to writing and editing the manuscript.

## Supporting information

Supporting Information

## Data Availability

The data that support the findings of this study are available from the corresponding author upon reasonable request.
