# Supplementary material for: Odd and Even Numbered Ferric Wheels
Source: Adv Sci (Weinh). 2023 Aug 27;10(31):2304553. doi: 10.1002/advs.202304553 (PMC10625049; doi:10.1002/advs.202304553)
Supplement: Supplementary file 1 — Supporting Information [file ADVS-10-2304553-s001.pdf]

ADVANCED SCIENCE
Open Access

Supporting Information

for *Adv. Sci.*, DOI 10.1002/advs.202304553

Odd and Even Numbered Ferric Wheels

Daniel J. Cutler, Angelos B. Canaj, Mukesh K. Singh, Gary S. Nichol, David Gracia, Hiroyuki Nojiri, Marco Evangelisti*, Jürgen Schnack* and Euan K. Brechin**

Experimental Procedures

Synthesis of [$Fe^{III}_{11}Zn^{II}_{4}(tea)_{10}(teaH)(OMe)Cl_8$] (1)

$FeCl_3$ (1 mmol, 0.162 g) and $Zn(ClO_4)_2·6H_2O$ (1 mmol, 0. 372 g) were dissolved in a 50:50 mixture of MeCN and MeOH (25 ml). Triethanolamine $teaH_3$ (1.5 mmol, 0.2 ml) and NEt_3 (3 mmol, 0.42 ml) were then added dropwise into the reaction mixture. The cloudy yellow solution was stirred at room temperature for 12 hours. After 12 hours the reaction mixture produces a yellow-gold precipitate which was filtered. The filtered solution was left to stand in a capped 30 ml vial. After 3-4 days of standing yellow rod-shaped crystals suitable for X-ray diffraction appeared at the bottom of vial. Elemental analysis (% C H N) calculated (found) for $C_{67}H_{148}Cl_8Fe_{11}N_{11}O_{40}Zn_4$: C 27.68 (27.43), H 5.13 (4.91), N 5.30 (5.17). Metal analysis by ICP-OES (%wt Fe, Zn) calculated (found): Fe 21.94 (21.52) Zn 9.34 (9.35).

Synthesis of [$Fe_{12}Zn_4(tea)_{12}Cl_8$] (2)

$FeCl_3$ (1 mmol, 0.162 g) and $Zn(ClO_4)_2·6H_2O$ (1 mmol, 0. 372 g) were dissolved in a 50:50 mixture of MeCN and DMF (25 ml). $teaH_3$ (1.5 mmol, 0.2 ml) and NEt_3 (3 mmol, 0.42 ml) were then added dropwise. The cloudy orange solution was stirred at room temperature for 12 hours producing an orange precipitate. The reaction mixture was filtered and the clear solution left to stand in a capped 30 ml vial. After 4-5 days of standing dark yellow rod-shaped crystals suitable for X-ray diffraction formed at the bottom of the vial. Elemental analysis (% C H N) calculated (found) for $C_{72}H_{140}Cl_8Fe_{12}N_{16}O_{36}Zn_4$: C 31.93 (32.48), H 5.24 (5.53), N 6.77 (7.08). Metal analysis by ICP-OES (%wt Fe Zn) calculated (found): Fe 20.25 (19.88) Zn 7.90 (7.53).

Single crystal X-ray crystallography

Diffraction data for compound **1** were collected on a Bruker APEX-II CCD diffractometer. The crystals were kept at a steady temperature of T = 100.4 K throughout data collection using an Oxford Cryosystems Cryostream. The dataset was truncated at 1 Å due to rapidly rising values of R_{int} at higher resolution. The structure was solved using ShelXT and refined with ShelXL interfaced through Olex2.[1-3] Diffraction data for compound **2** were collected on a Rigaku Oxford Diffraction Xcalibur diffractometer. The crystals were kept at a steady temperature of T = 120.0 K throughout data collection using an Oxford Cryosystems Cryostream. The structure was solved using ShelXT and refined with ShelXL interfaced through Olex2.[1-3] CCDC 2202758-2202759.

Powder X-ray diffraction

Diffraction data for compounds **1** and **2** were collected on polycrystalline powders using a Bruker D8 ADVANCE with Cu radiation at 40 kV, 40 mA and a Johansson monochromator, 2 mm divergence slit and 2.5 degree Soller slits on the incident beam side, LynxEye detector and Bruker DIFFRAC software. Diffraction data were measured from 2θ = 5° - 40°; step size, 0.0101°. Freshly prepared crystalline powders of the samples were loaded into borosilicate capillaries with a 0.7 mm inside diameter and measured while spinning.

Magnetometry

Magnetic susceptibility data were collected on freshly prepared polycrystalline powders on a Quantum Design Dynacool PPMS equipped with a 9 T magnet in the temperature range 2 - 300 K. The samples were added in Quantum Design VSM Powder Sample Holders (P125E) with eicosane present and then transferred to PPMS brass half-tube sample holders. Diamagnetic corrections from the holders and eicosane were applied. In addition, diamagnetic corrections were applied to the observed paramagnetic susceptibilities using Pascal's constants.

Low-temperature, high field magnetisation data were measured by the use of a conventional inductive probe in pulsed magnetic fields, where the temperature reached as low as 0.4 K. The maximum field reached was 32.5 T. Polycrystalline samples with a typical mass of 10 mg were mounted in a capillary tube made of polyimide. The sample, which was not fixed within the sample tube, was aligned along the magnetic field direction. Magnetisation curves were found to be identical after we applied the magnetic field several times due to the saturation of the orientation effect.

Heat Capacity

Heat capacity measurements were carried out using a Quantum Design PPMS in the temperature range 2 - 50 K. The polycrystalline samples were in the form of a thin pressed pellet (ca. 1 mg), thermalized by ca. 0.2 mg of Apiezon N grease, whose contribution was subtracted by using a phenomenological expression.

Computational Details

We have used Density Functional Theory (DFT) in the Gaussian 09 suite[4] to compute the magnetic exchange coupling constants (*J*) for **1**-**2** on tri- and tetrametallic model complexes created from the crystal structures of **1** and **2**. These are models **1M1**-**1M5** for **1** and models **2M1**-**2M2** for **2** (Figures S9-10). Models **1M1**-**1M4** and **2M1**-**2M2** are trimetallic models with terminal Ga^{III} ions employed to maintain the same electronic environment. In model **1M5**, four Fe^{III} centres and two terminal Ga^{III} ions are used. Noodleman's broken symmetry approach, [5] a reliable tool for estimating magnetic exchange coupling, has been employed to estimate the magnetic exchange interactions. For the trimetallic models (**1M1**-**1M4** and **2M1**-**2M2**) we have computed one high spin configuration with all three Fe^{III} spins aligned parallel (S = 15/2) and three broken symmetry configurations with one of the Fe^{III} spins aligned antiparallel to other two (S = 5/2). For **1M5** we have computed one high spin configuration with all four Fe^{III} spins aligned parallel (S = 10), two broken symmetry configurations with one of the Fe^{III} spins aligned antiparallel (S = 5), and three broken symmetry configurations with two of the Fe^{III} spins aligned anti-parallel (S = 0). All spin configurations are summarised in Tables S7-S8. The errors associated with all the estimated magnetic exchange values are found to be less than 0.1%. We have employed the hybrid B3LYP functional[6] with the TZV basis set for Fe, the SVP basis set for Ga, Zn, O, N and the SV basis set for Cl, C and H.[7]

Table S1. *Crystallographic details of **1** and **2**.*

**Compound**	**1**	**2**
**Formula**	$C_{67}H_{148}Cl_8Fe_{11}N_{11}O_{40}Zn_4$	$C_{96}Cl_8Fe_{12}H_{178}N_{24}O_{37}Zn_4$
$D_{calc.}$**/ g cm**$^{-3}$	1.494	1.477
m**/mm**$^{-1}$	2.155	1.967
**Formula Weight**	2907.39	3321.76
**Colour**	yellow	dark yellow
**Shape**	rod-shaped	rod-shaped
**Size/mm**^{3}	0.18×0.06×0.04	0.34×0.11×0.10
T**/K**	100.40	120.00
**Crystal System**	monoclinic	tetragonal
**Space Group**	$P2_1/n$	$I4_1/a$
a**/Å**	18.5721(9)	35.7793(4)
b**/Å**	32.2714(16)	35.7793(4)
c**/Å**	21.8429(11)	11.6698(3)
$\alpha/^{\circ}$	90	90
$\beta/^{\circ}$	99.171(2)	90
$\gamma/^{\circ}$	90	90
**V/Å**^{3}	12924.1(11)	14939.2(5)
Z	4	4
Z'	1	0.25
**Wavelength/Å**	0.71073	0.71073
**Radiation type**	Mo K_{α}	Mo K_{α}
$Q_{min}/^{\circ}$	2.116	3.340
$Q_{max}/^{\circ}$	20.831	25.671
**Measured Refl's.**	303804	74482
**Indep't Refl's**	13524	7077
**Refl's I≥2** s**(I)**	11513	6165
R_{int}	0.0896	0.0573
**Parameters**	1272	457
**Restraints**	19	127
**Largest Peak**	0.777	1.006
**Deepest Hole**	-0.569	-0.525
**GooF**	1.019	1.140
wR_2 **(all data)**	0.0707	0.1439
wR_2	0.0670	0.1394
R_1 **(all data)**	0.0396	0.0761
R_1	0.0307	0.0663

Table S2. *Bond valence sum calculations for the metal ions in* ***1*** *and* ***2****.*

**1**		**2**	
**Atom**		**Atom**	
**Zn01**	2.04	**Zn1**	2.12
**Zn03**	2	**Fe1**	3.00
**Zn04**	1.96	**Fe2**	2.98
**Fe01**	2.91	**Fe3**	2.92
**Fe02**	3.223		
**Fe03**	3.05		
**Fe04**	2.9		
**Fe05**	2.95		
**Fe06**	2.89		
**Fe07**	3.01		
**Fe08**	3.02		
**Fe09**	2.95		
**Fe10**	2.93		
**Fe11**	2.98		

Table S3. *Selected bond angles in* ***1.***

**Atom**	**Atom**	**Atom**	**Angle (°)**	**Atom**	**Atom**	**Atom**	**Angle (°)**
Fe1	O1	Fe11	107.79(13)	Fe6	O16	Fe5	104.64(12)
Fe1	O1	Fe11	107.79(13)	Fe6	O18	Fe7	103.97(12)
Fe1	O1	Fe11	107.79(13)	Fe7	O19	Fe6	105.69(12)
Fe2	O3	Fe1	106.65(13)	Fe7	O20	Fe8	103.48(12)
Fe2	O4	Fe1	104.05(13)	Fe8	O21	Fe7	105.22(13)
Fe2	O6	Fe3	102.79(13)	Fe8	O24	Fe9	102.32(13)
Fe3	O7	Fe2	102.71(13)	Fe9	O25	Fe8	105.21(13)
Fe4	O9	Fe3	106.23(13)	Fe10	O26	Fe9	106.40(13)
Fe3	O10	Fe4	101.43(12)	Fe10	O27	Fe9	106.00(13)
Fe5	O12	Fe4	106.76(13)	Fe11	O29	Fe10	104.32(13)
Fe5	O13	Fe4	106.49(13)	Fe10	O30	Fe11	104.02(13)
Fe5	O15	Fe6	103.70(12)	Fe11	O33	Fe1	105.85(13)

Table S4. *Selected bond lengths in* ***1****.*

Atom	Atom	Length (Å)	Atom	Atom	Length (Å)
Fe1	O1	1.983(3)	Fe6	O18	2.008(3)
Fe1	O2	1.864(3)	Fe6	O19	2.025(3)
Fe1	O3	2.028(3)	Fe6	N6	2.244(3)
Fe1	O4	2.041(3)	Fe7	O18	2.030(3)
Fe1	O33	2.076(3)	Fe7	O19	1.966(3)
Fe1	N1	2.320(4)	Fe7	O20	1.968(3)
Fe2	O3	1.957(3)	Fe7	O21	1.991(3)
Fe2	O4	2.013(3)	Fe7	O22	1.975(3)
Fe2	O5	1.976(3)	Fe7	N7	2.244(3)
Fe2	O6	1.947(3)	Fe8	O20	2.031(3)
Fe2	O7	2.023(3)	Fe8	O21	1.961(3)
Fe2	N2	2.176(4)	Fe8	O23	1.965(3)
Fe3	O6	2.034(3)	Fe8	O24	1.977(3)
Fe3	O7	1.961(3)	Fe8	O25	1.997(3)
Fe3	O8	1.949(3)	Fe8	N8	2.235(4)
Fe3	O9	1.994(3)	Fe9	O24	2.077(3)
Fe3	O10	1.989(3)	Fe9	O25	1.979(3)
Fe3	N3	2.230(4)	Fe9	O26	2.016(3)
Fe4	O9	1.977(3)	Fe9	O27	2.019(3)
Fe4	O10	2.113(3)	Fe9	O28	1.891(3)
Fe4	O11	1.875(3)	Fe9	N9	2.261(4)
Fe4	O12	2.010(3)	Fe10	O26	1.985(3)
Fe4	O13	2.038(3)	Fe10	O27	1.993(3)
Fe4	N4	2.318(4)	Fe10	O29	2.031(3)
Fe5	O12	2.010(3)	Fe10	O30	1.984(3)
Fe5	O13	1.989(3)	Fe10	O31	1.964(3)
Fe5	O14	1.970(3)	Fe10	N10	2.306(4)
Fe5	O15	1.982(3)	Fe11	O1	2.025(3)
Fe5	O16	2.028(3)	Fe11	O29	1.976(3)
Fe5	N5	2.262(4)	Fe11	O30	2.031(3)
Fe6	O15	2.040(3)	Fe11	O32	1.959(3)
Fe6	O16	1.969(3)	Fe11	O33	1.981(3)
Fe6	O17	1.971(3)	Fe11	N11	2.259(4)

Table S5. *Selected bond lengths in* ***2****.*

**2**		
**Atom**	**Atom**	**Distance (Å)**
Fe1	O1	1.978(3)
Fe1	O2	2.028(4)
Fe1	O3	1.984(4)
Fe1	O4	1.952(4)
Fe1	O9	1.990(3)
Fe1	N1	2.213(5)
Fe2	O2	1.981(4)
Fe2	O4	2.027(4)
Fe2	O5	1.955(4)
Fe2	O6	1.982(3)
Fe2	O7	1.977(8)
Fe2	O7A	2.020(9)
Fe2	N2	2.258(6)
Fe3	O3	2.088(3)
Fe3	O6	2.020(4)
Fe3	O7	2.040(8)
Fe3	O7A	2.022(9)
Fe3	O8	1.867(5)
Fe3	O9	1.971(4)
Fe3	N3	2.290(5)

Table S6. *Selected bond angles in* ***2****.*

**2**			
**Atom**	**Atom**	**Atom**	**Angle (°)**
**Fe1**	O3	Fe3[1]	101.48(15)
**Fe1**	O4	Fe2	103.29(16)
**Fe2**	O6	Fe3	105.77(15)
**Fe2**	O7A	Fe3	104.3(4)
**Fe2**	O2	Fe1	102.22(16)
**Fe3**	O9	Fe1[2]	105.50(16)

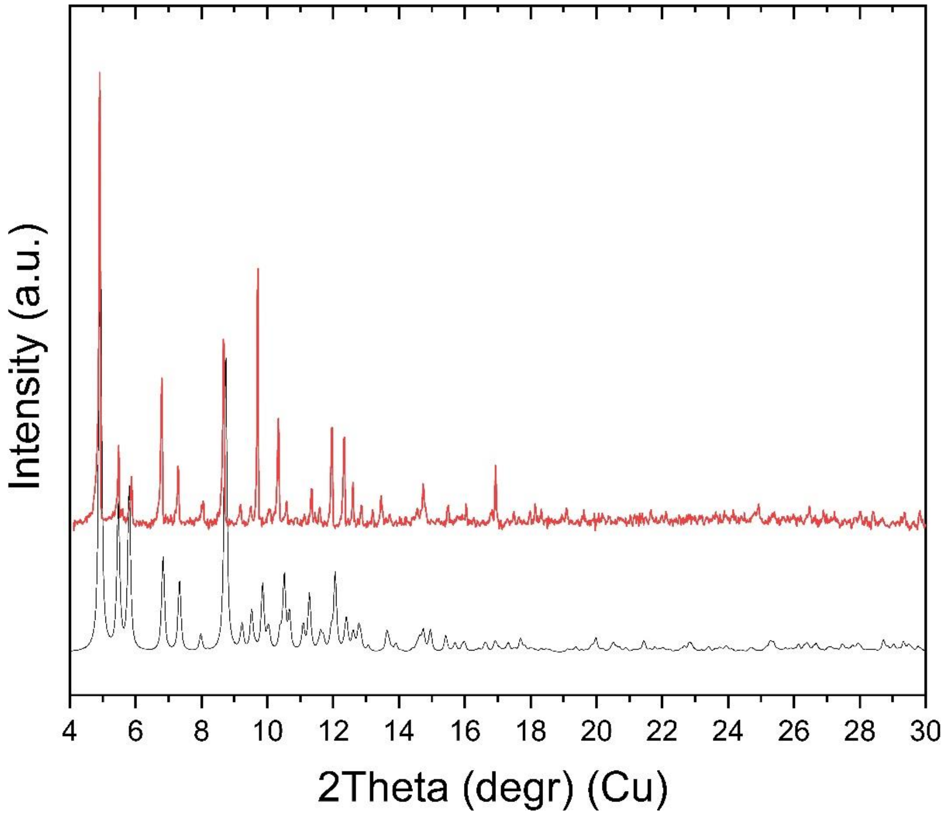

Figure S1. Powder X-ray diffraction pattern of **1**. Experimental (red), predicted (grey). Crystals are stable outside the mother liquor and *in vacuo*.

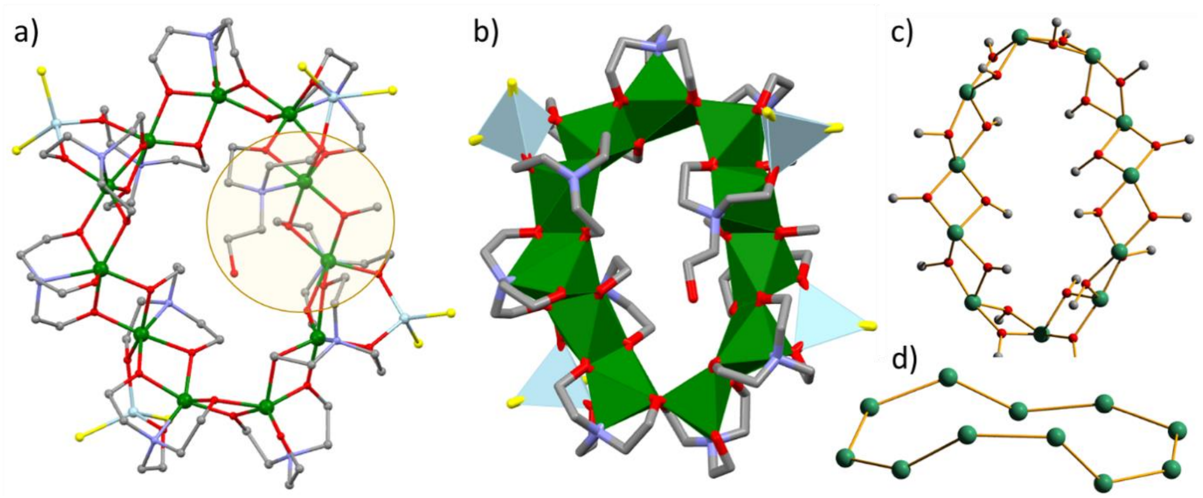

Figure S2. Alternative views of the molecular structure of complex **1** in (a) ball and stick and (b) polyhedral formats. (c) The metal-oxygen magnetic core. (d) The metallic skeleton highlighting the non-planar arrangement of Fe^{III} ions. Colour code: Fe = green, Zn = pale blue, O = red, N = blue, C = grey, Cl = yellow. H atoms omitted. The highlighted section in a) shows the Fe6-Fe7 unit containing the μ-OMe ligand and the non-bonded arm of the teaH ligand.

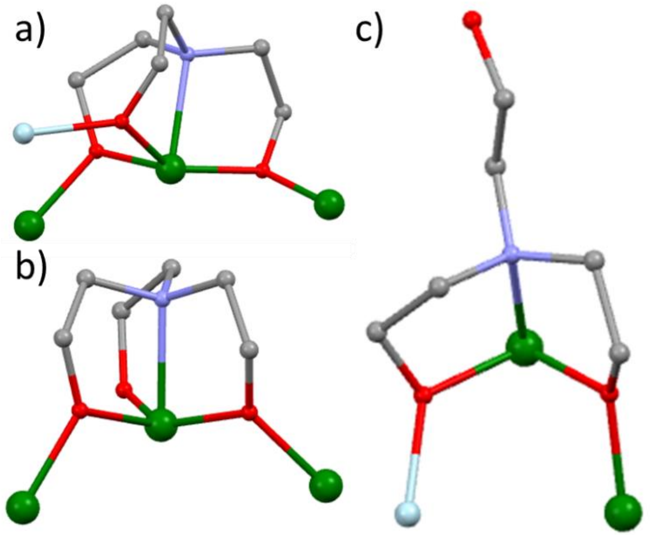

Figure S3. Bridging modes of the triethanolamine ligands. a) η^2, η^2, η^2, μ_4-tea^{3-}, [Fe_3Zn]. b) η^2, η^2, η^1, μ_3-tea^{3-}, [Fe_3]. c) η^2, η^2, μ_3-Htea^{3-}, [Fe_2Zn]. Colour code: Fe = green, Zn = pale blue, O = red, N = blue, C = grey. H atoms omitted. All three bridging modes are present in **1**, but only two (a, b) are present in **2**.

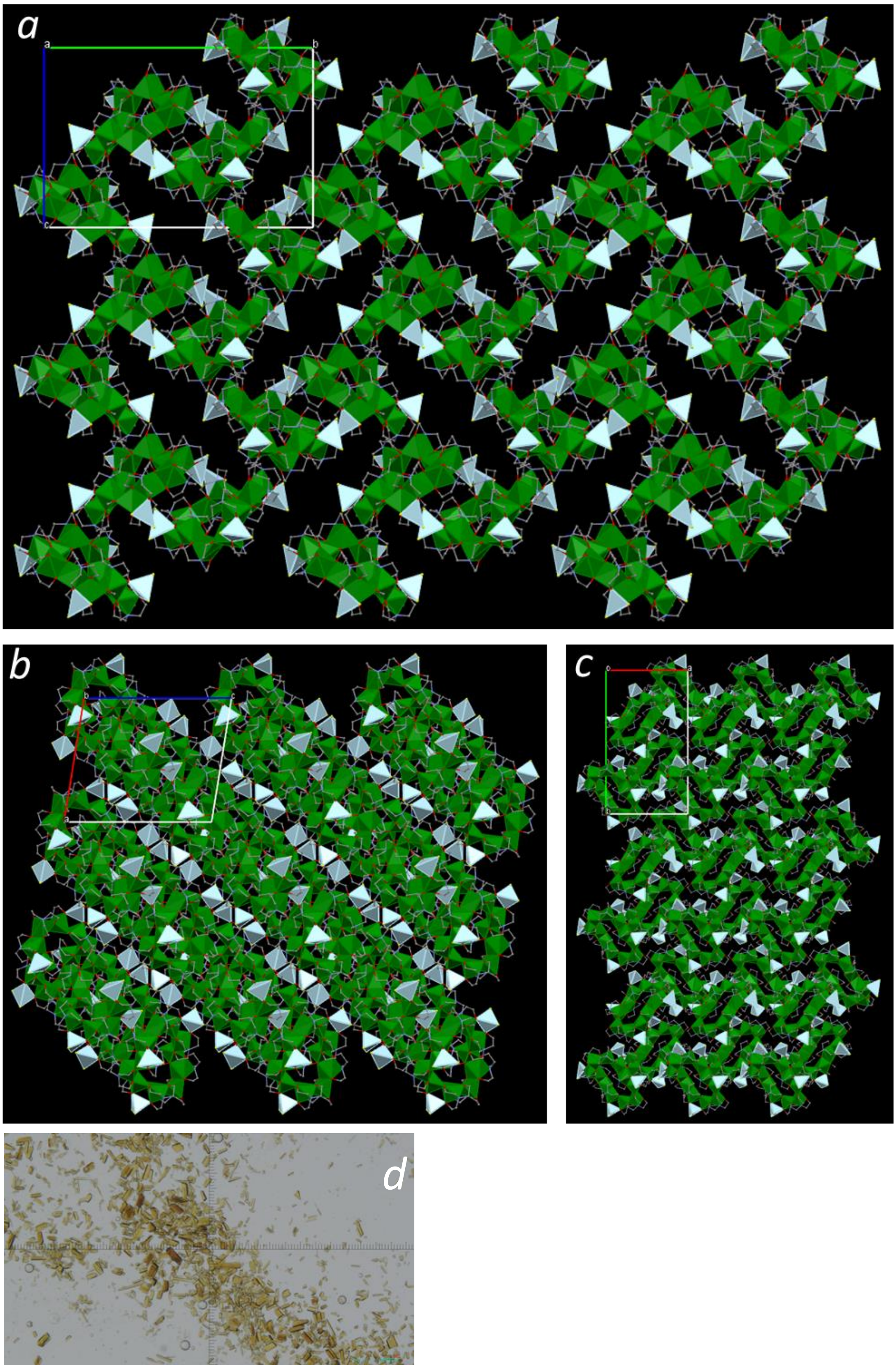

Figure S4. The packing of **1** in the extended staructure as viewed down the *a*-, *b*- and *c*-axis, respectively. The clusters are shown in polyhedral format. Colour code: Fe = green, Zn = pale blue, O = red, N = blue, C = grey. H atoms omitted. d) Microscope image of the crystals of **1**.

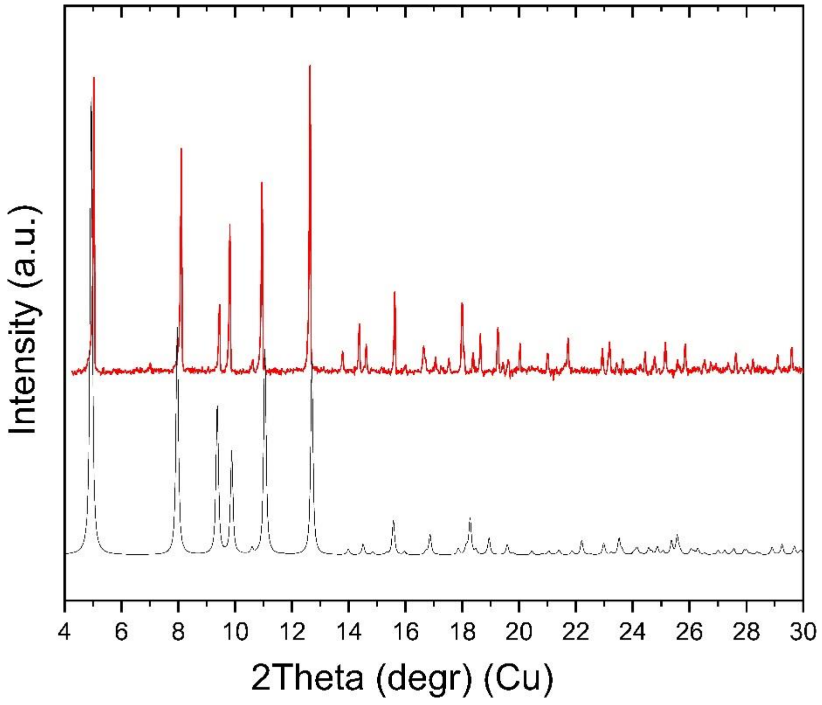

Figure S5. Powder X-ray diffraction pattern of **2**. Experimental (red), predicted (grey). Crystals are stable outside the mother liquor and *in vacuo*.

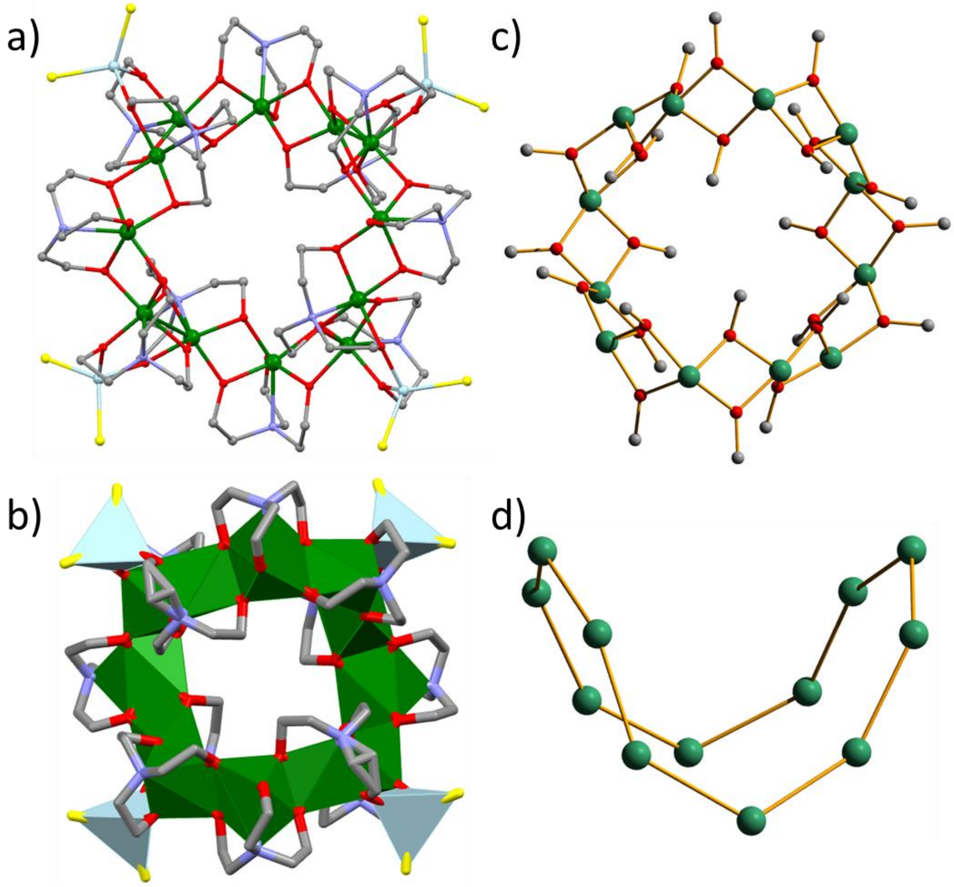

Figure S6. Alternative views of the molecular structure of complex **2** in (a) ball and stick, and (b) polyhedral formats. (c) The metal-oxygen magnetic core. (d) The metallic skeleton highlighting the bowl- or U-shaped arrangement of Fe^{III} ions. Colour code: Fe = green, Zn = pale blue, O = red, N = blue, C = grey, Cl = yellow. H atoms omitted.

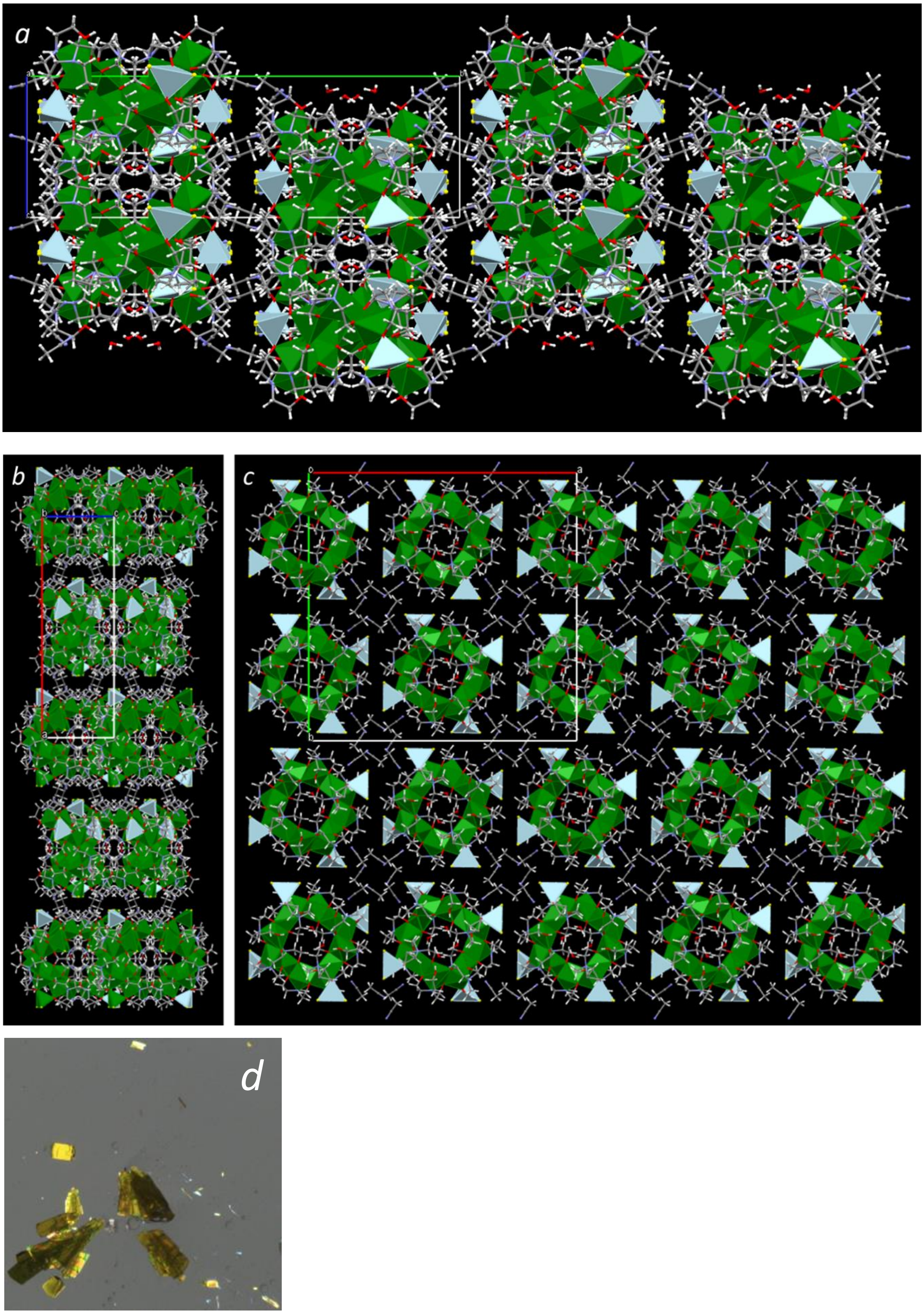

Figure S7. The packing of **2** in the extended structure as viewed down the *a*-, *b*- and *c*-axis, respectively. The clusters are shown in polyhedral format. Colour code: Fe = green, Zn = pale blue, O = red, N = blue, C = grey. H atoms omitted. d) Microscope image of the crystals of **2**.

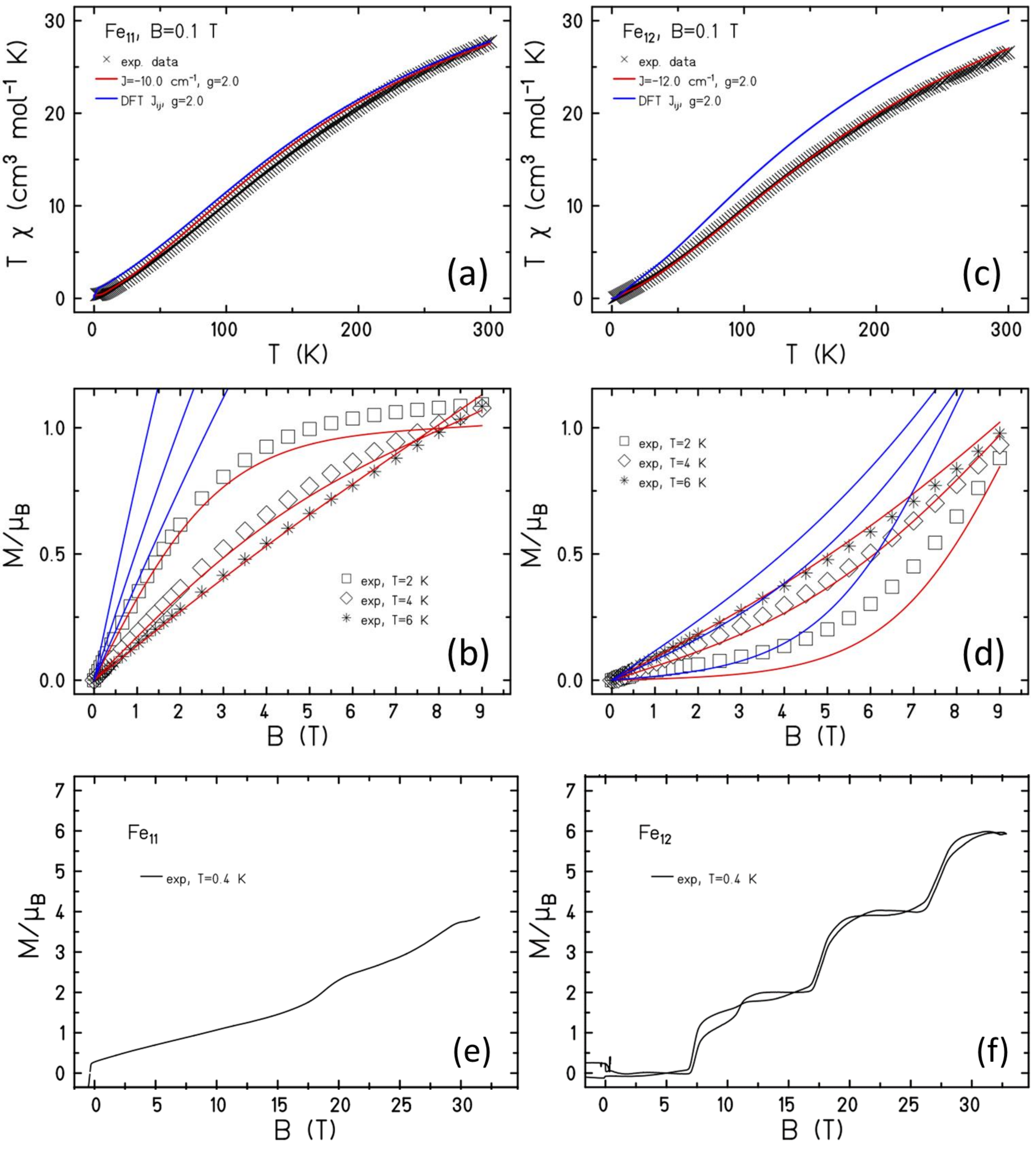

Figure S8. Magnetic susceptibility and magnetisation data for **1** (a, b) and **2** (c, d). The solid red lines are simulations of the experimental data with J = -10.0 cm^{-1} and J = -12.0 cm^{-1}, respectively. The solid blue lines are simulations using the DFT derived J values. See the main text for details. High field magnetisation data (e, f) measured at T = 0.4 K for **1** and **2**.

Table S7. Spin configurations employed for the trimetallic models **1M1**-**1M4** and **2M1**-**2M2** derived from the crystal structures of **1-2**.

	S value	Fe1	Fe2	Fe3
HS	15/2	↑	↑	↑
BS-1	5/2	↓	↑	↑
BS-2	5/2	↑	↓	↑
BS-3	5/2	↑	↑	↓

Table S8. Spin configurations employed for the tetrametallic model **1M5** derived from the crystal structure of **1**.

	S value	Fe1	Fe2	Fe3	Fe4
HS	10	↑	↑	↑	↑
BS-1	5	↓	↑	↑	↑
BS-2	5	↑	↑	↑	↓
BS-3	0	↓	↓	↑	↑
BS-4	0	↓	↑	↑	↓
BS-5	0	↑	↓	↓	↑

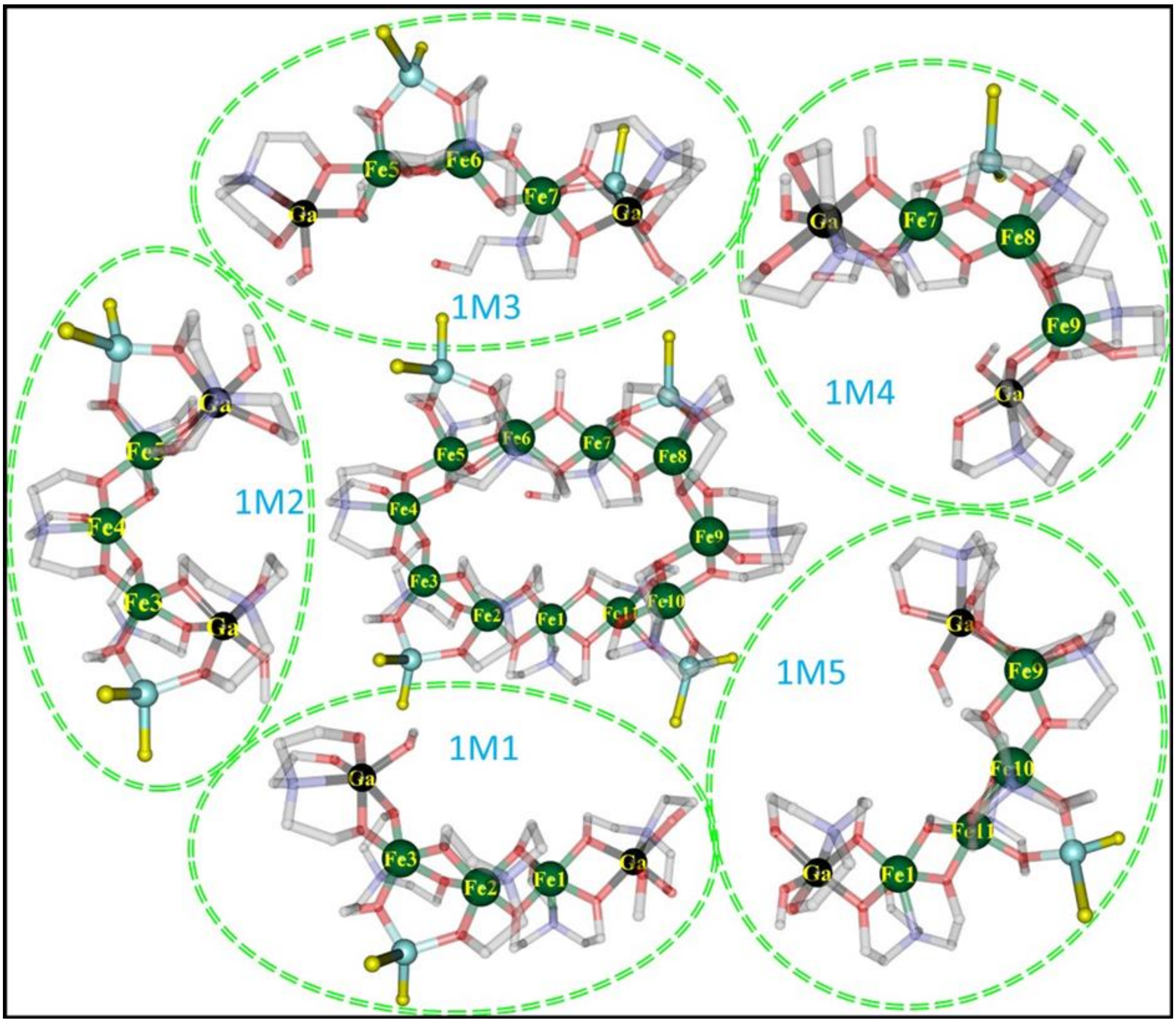

Figure S9. The model complexes **1M1**-**1M5** (dashed green circles) employed to calculate the magnetic exchange interactions in **1** (centre). Colour Code: Fe, dark-green; Ga, black; Zn, cyan; Cl, yellow; O, red; N, blue; C, grey. H atoms omitted for clarity. The terminal Fe^{III} centres in all model complexes have been replaced with Ga^{III} ions in order to keep the electronic environment around the Fe^{III} centres the same as in **1**.

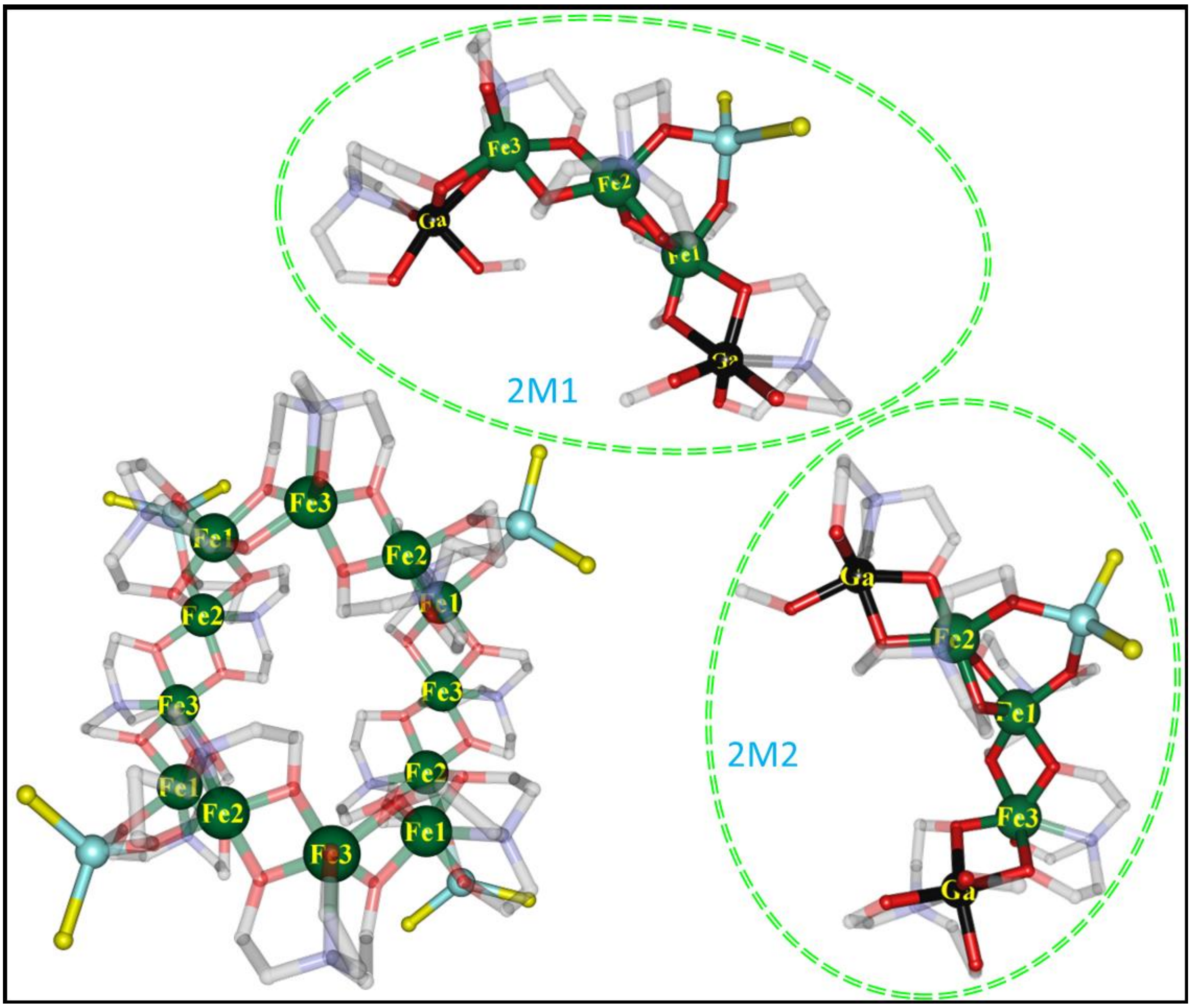

Figure S10. The model complexes **2M1**-**2M2** (dashed green circles) employed to calculate the magnetic exchange interactions in **2** (lower left). Colour Code: Fe, dark-green; Ga, black; Zn, cyan; Cl, yellow; O, red; N, blue; C, grey. H atoms omitted for clarity. The terminal Fe^{III} centres in all model complexes have been replaced with Ga^{III} ions in order to keep the electronic environment around the Fe^{III} centres the same as in **2**.

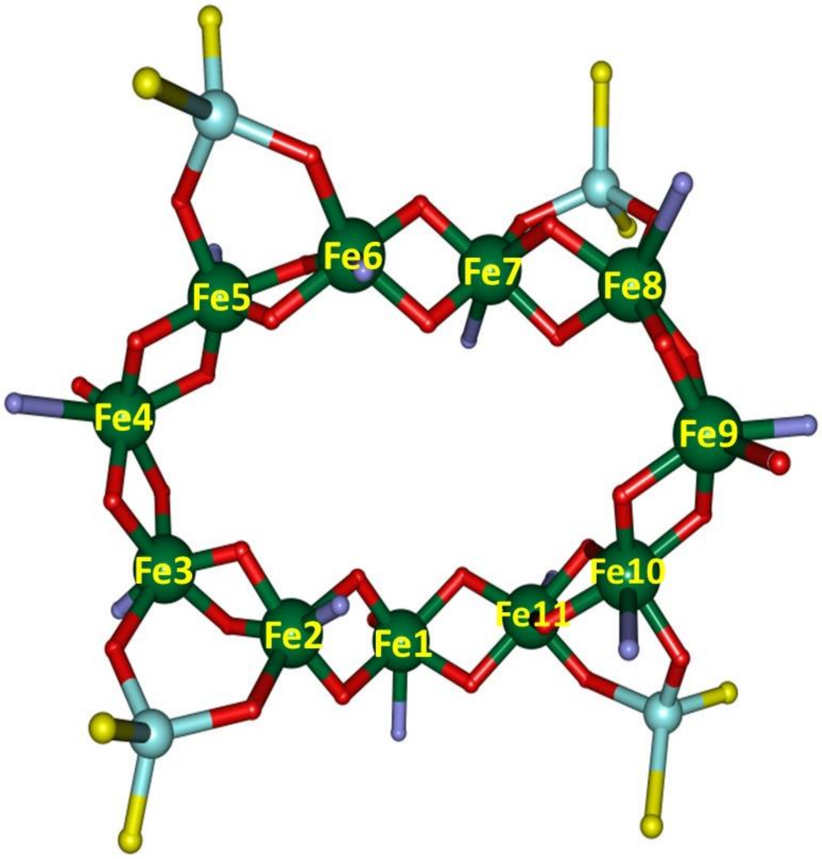

	J cm^{-1}
$J_{Fe1\text{-}Fe2}$	-6.6
$J_{Fe2\text{-}Fe3}$	-10.2
$J_{Fe3\text{-}Fe4}$	-9.3
$J_{Fe4\text{-}Fe5}$	-12.7
$J_{Fe5\text{-}Fe6}$	-9.2
$J_{Fe6\text{-}Fe7}$	-9.8
$J_{Fe7\text{-}Fe8}$	-12.5
$J_{Fe8\text{-}Fe9}$	-9.7
$J_{Fe9\text{-}Fe10}$	-11.3
$J_{Fe10\text{-}Fe11}$	-3.5
$J_{Fe1\text{-}Fe11}$	-12.8

Figure S11. The metal-oxygen core of **1** with the tabulated J_{DFT} values calculated for each bridge labelled. Colour Code: Fe, green; Zn, cyan; Cl, yellow; O, red; N, blue; C, grey. H atoms omitted for clarity.

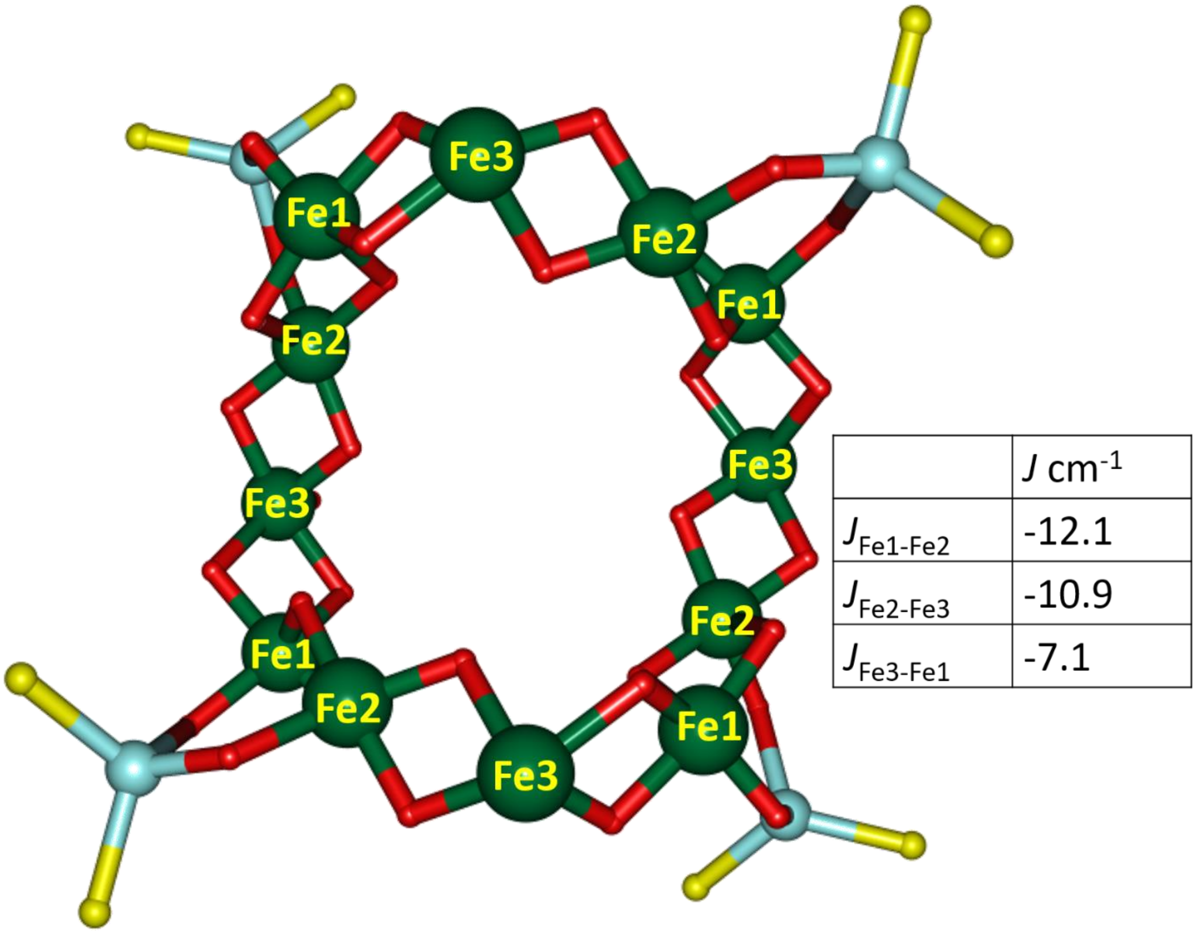

	J cm^{-1}
$J_{\text{Fe1-Fe2}}$	-12.1
$J_{\text{Fe2-Fe3}}$	-10.9
$J_{\text{Fe3-Fe1}}$	-7.1

Figure S12. The metal-oxygen core of **2** with the tabulated J_{DFT} values calculated for each bridge labelled. Colour Code: Fe, green; Zn, cyan; Cl, yellow; O, red; N, blue; C, grey. H atoms omitted for clarity.

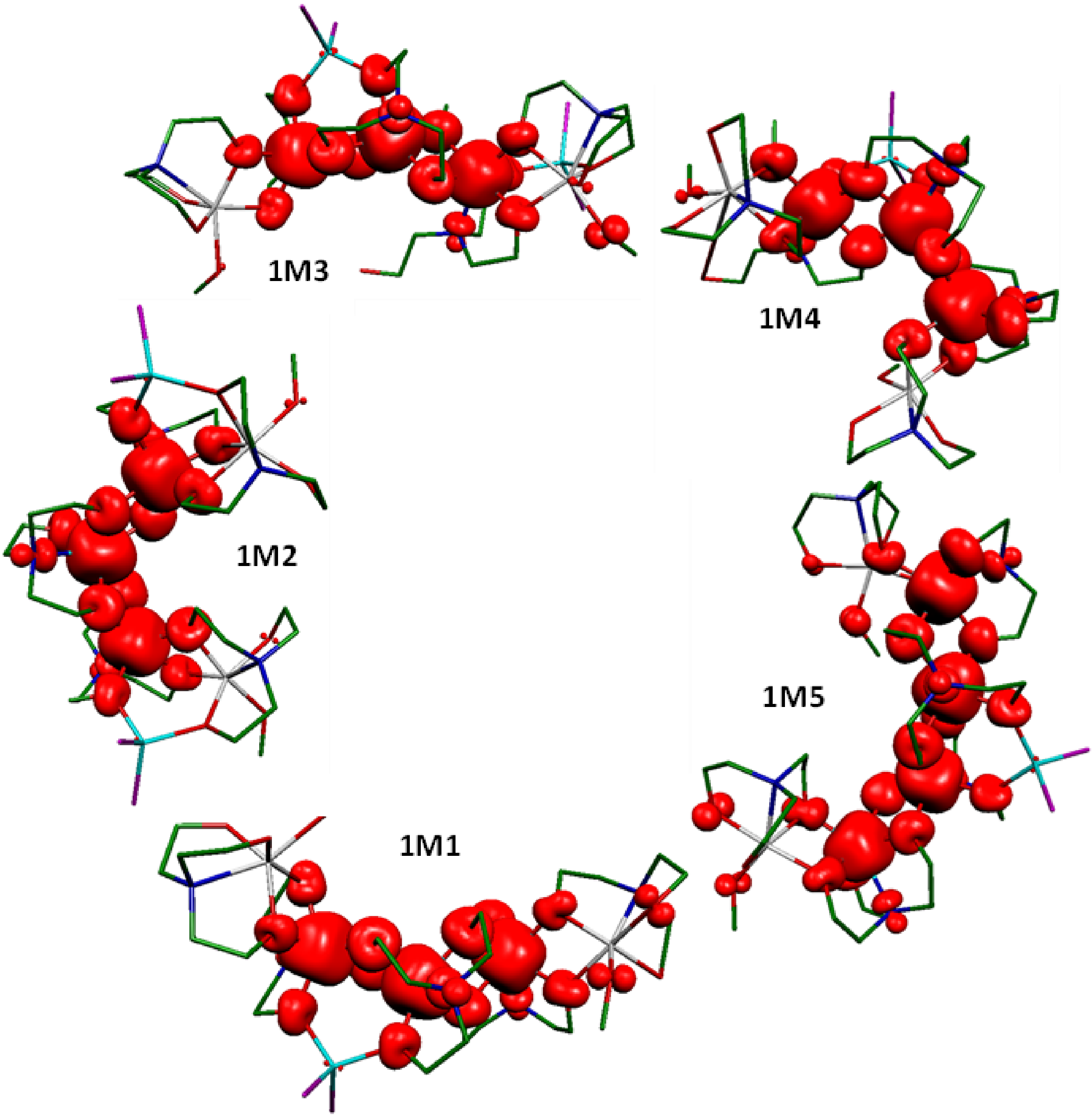

Figure S13. DFT computed spin density plots for models **1M1-1M5** indicating a strong spin-delocalization mechanism for the magnetic exchange. The isodensity surface for all the spin density plots are corresponding to 0.005 e bohr^{-3}.

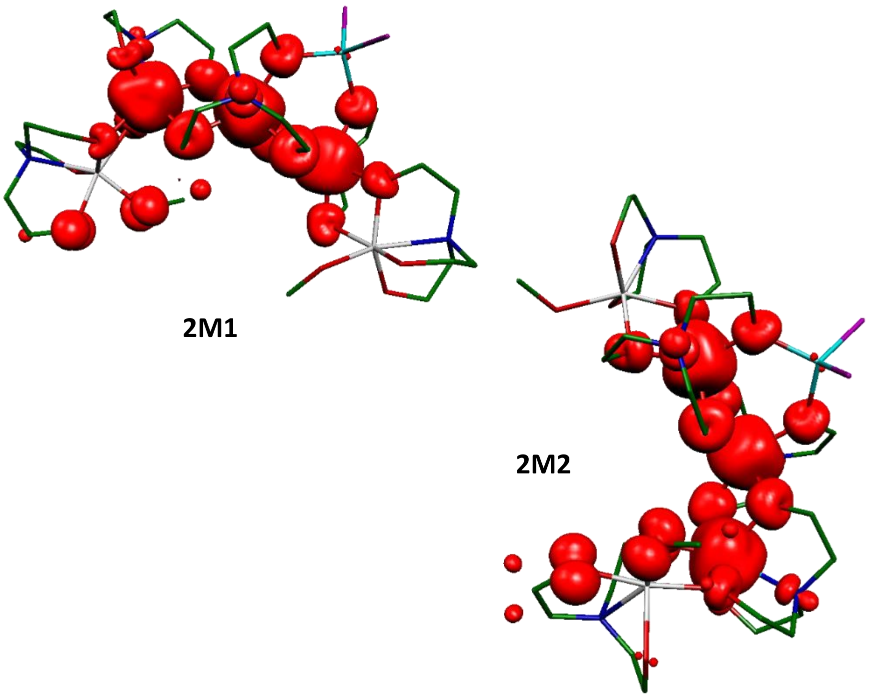

Figure S14. DFT computed spin density plots for models **2M1-2M2** indicating a strong spin-delocalization mechanism for the magnetic exchange. The isodensity surface for all the spin density plots are corresponding to 0.005 e bohr^{-3}.

References

[1] O. V. Dolomanov, L. J. Bourhis, R. J. Gildea, J. A. K. Howard, H. Puschmann, Olex2: A complete structure solution, refinement and analysis program, *J. Appl. Cryst*. **2009**, *42*, 339-341.

[2] G. M. Sheldrick, Crystal structure refinement with ShelXL, *Acta Cryst*. **2015**, *C71*, 3-8.

[3] G. M. Sheldrick, ShelXT-Integrated space-group and crystal-structure determination, *Acta Cryst*. **2015**, *A71*, 3-8.

[4] M. J. Frisch, G. W. Trucks, H. B. Schlegel, G. E. Scuseria, M. A. Robb, J. R. Cheeseman, G. Scalmani, V. Barone, B. Mennucci, G. A. Petersson, H. Nakatsuji, M. Caricato, X. Li, H. P. Hratchian, A. F. Izmaylov, J. Bloino, G. Zheng, J. L. Sonnenberg, M. Hada, M. Ehara, K. Toyota, R. Fukuda, J. Hasegawa, M. Ishida, T. Nakajima, Y. Honda, O. Kitao, H. Nakai, T. Vreven, J. A. Montgomery, J. E. Peralta, F. Ogliaro, M. Bearpark, J. J. Heyd, E. Brothers, K. N. Kudin, V. N. Staroverov, R. Kobayashi, J. Normand, K. Raghavachari, A. Rendell, J. C. Burant, S. S. Iyengar, J. Tomasi, M. Cossi, N. Rega, J. M. Millam, M. Klene, J. E. Knox, J. B. Cross, V. Bakken, C. Adamo, J. Jaramillo, R. Gomperts, R. E. Stratmann, O. Yazyev, A. Austin, J. R. Cammi, C. Pomelli, J. W. Ochterski, R. L. Martin, K. Morokuma, V. G. Zakrzewski, G. A. Voth, P. Salvador, J. J. Dannenberg, S. Dapprich, A. D. Daniels, Ö. Farkas, J. B. Foresman, J. V. Ortiz, J. Cioslowski, D. J. Fox, *Gaussian 09, Revision E.01*, Wallingford CT **2013**.

[5] L. Noodleman, *J. Chem. Phys.* **1981**, *74*, 5737-5743.

[6] a) A. D. Becke, *Phys. Rev. A*, **1988**, *38*, 3098-3101; b) A. D. Becke, *J. Chem. Phys.* **1993,** *98*, 5648-5652; c) C. Lee, W. Yang, R. G. Parr, *Phys. Rev. B: Condens. Matter Mater. Phys.* **1988**, *37*, 785.

[7] a) A. Schafer, H. Horn, R. Ahlrichs, *J. Chem. Phys.* **1992**, *97*, 2571-2577; b) A. Schäfer, C. Huber, R. Ahlrichs, *J. Chem. Phys.* **1994**, *100*, 5829-5835; c) G. E. Scuseria, H. F. Schäfer, *J. Chem. Phys.* **1989**, *90*, 3700-3703.
